# Community awareness of diet needs associated with hypertension and type 2 diabetes mellitus in Hatcliffe, Zimbabwe

**DOI:** 10.1186/s12889-019-8030-4

**Published:** 2019-12-16

**Authors:** Lonestar Lazarus Gonde, Moses John Chimbari

**Affiliations:** University of KwaZulu Natal, College of Health Sciences, School of Nursing and Public Health, Durban, 4041 South Africa

**Keywords:** Hypertension, Type 2 diabetes mellitus, Awareness, Dietary needs, Zimbabwe

## Abstract

**Background:**

Diet is an important modifiable risk factor for non-communicable diseases (NCDs) like hypertension (HTN) and type 2 diabetes mellitus (T2DM). A NCD is a disease that cannot be transmitted from person to person. Dietary risk factors account for 5.8% of all-cause mortality in Sub-Saharan Africa (SSA). There has been an increase in the consumption of ‘westernized ‘diets in SSA. The westernized diets consumed in low-income countries are usually high in salt content, fatty, processed and fast foods; and hence accelerate the development of HTN and T2DM. Previous studies carried out in Zimbabwe showed low levels of knowledge and awareness of HTN and T2DM; and the dietary needs for patients with those conditions. The aim of this study was to explore the dietary habits and awareness of HTN and T2DM of both males and females in a high-density area (HDA) of Zimbabwe.

**Methods:**

We conducted household-based cross-sectional study in a high density area of Hatcliffe, which has a population of close to 50,000 residents. Face to face interviews were conducted using hand-held mobile devices loaded with KoBo Toolbox. We selected two consenting adults, a male and female, from every fourth household in selected areas of Hatcliffe.

**Results:**

In this study all the 492 participants that were interviewed responded. Eighty eight point 6% (88.6%) of the participants in the study did not know if they were hypertensive or not. In addition, 91.7% of the participants had never voluntarily checked for hypertension. Similarly, 97.6% of the participants did not know if they had T2DM or not. Ninety eight percent (98%) of the participants had not voluntarily checked their blood glucose level.

**Conclusions:**

The majority of the participants in the study were not aware if they had HTN or T2DM. The participants in the study perceived that the salt they consume is the right quantity. There is a high consumption of vegetable oil in most meals prepared.

## Background

Globally the prevalence of non-communicable diseases (NCDs) has drastically increased in the past decade [1]. A 68% increase in deaths as a result of NCDs from 1990 to 2013 in sub-Saharan Africa (SSA) has been reported [2]. Consistent with global trends most SSA countries have for the past 20 years been experiencing an increase in cases of hypertension (HTN) and Type 2 Diabetes Mellitus (T2DM) [3]. According to Lachat et al most low to medium income countries (LMIC) are not well equipped to fight the risk factors of NCDs [4]. The rise in the prevalence of HTN and T2DM has largely been due to an epidemiological and nutritional transition [5]. The high costs for treating HTN and T2DM exert more pressure on healthcare systems in SSA [6].

Diet is an important modifiable risk factor for NCDs like HTN and T2DM [7]. Dietary risk factors account for 5.8% of all-cause mortality in SSA [2]. There is lack of interventions for discouraging unhealthy eating habits in most LMIC [4]. Moreover, there has been a departure from eating the traditional staple diets in most SSA countries and increased preference for western diets that are risky. The westernized diets consumed in low-income countries are usually high in salt content, fatty, processed and fast foods; and hence accelerate the development of HTN and T2DM [4, 5]. The increase in cases of HTN and T2DM is therefore largely due to, among other factors, high salt intake, trans fats and taking excessive energy foods [3, 7].

The World Health Organization’s (WHO) Surveillance Harnessing Adopting Environment (SHAKE) Technical Package for Salt Reduction is a strategy designed to reduce salt consumption for its member countries. The WHO’s SHAKE Technical Package for Salt Reduction if implemented and operationalized within a country could reduce salt intake by people. Africans are known to consume large amounts of salt in their diet [8]. WHO had set targets for relative reduction of salt consumption to 30% but by 2014 none of the low-income countries had reported any significant reduction [9, 10]. Experts have recommended multisectoral approaches anchored on three pillars for the nationwide salt reduction. The pillars are i) production reformulation of consumables at an industrial level ii) educating and raising awareness of the consumers and iii) environmental changes [11]. Some cultural aspects and poverty in Africa are seen as the reason why the WHO’s (Dietary Approach to Stop Hypertension) (DASH) initiative cannot be implemented successfully [12]. An increase in the urban population has resulted in more salt consumption [11] .

In an effort to reduce and eliminate trans-fat consumption WHO developed and introduced the Reviewing, Promoting, Legislating, Assessment, Creating and Enforcing (REPLACE) strategy. WHO designed REPLACE as a tool to do away with ‘industrially-produced ***trans fat*** from the global food supply ’. REPLACE tool is anchored on the following pillars: (i) review of the source of the trans fats in food (ii) promotion of healthier oils (iii) legislation of laws that reject industrially manufactured trans fats (iv) assessment of foods to verify the amounts of trans fats (v) creation of awareness amongst population and stakeholders and (vi) enforcement of laws that aim to eliminate industrially manufactured trans fats.

Marshall et al*,* study on the Finnish Diabetes Prevention Program confirmed that reduction in the consumption of total and saturated fat reduces the risk of developing T2DM [13].

Pastakia et al recommend further investigations of dietary habits in SSA for better understanding of the development of diabetes [6]. In the SSA region, only South Africa has adopted the REPLACE strategy and is aiming to reduced levels of trans fats in all food stuffs to 2% by 2023 [14].

Zimbabwe is a signatory to the SHAKE strategy although implementation of the program is lagging behind. Knowledge has been documented as a key component to public health intervention such as SHAKE and REPLACE. Previous studies carried out in Zimbabwe among diabetic patients showed low levels of knowledge and awareness of this disease and the dietary needs for patients with the disease [15]. Hypertension is not prioritized in Zimbabwe and there is little awareness of appropriate diets for preventing or/and controlling it [16]. The paper explores the dietary habits and awareness of HTN and T2DM in a high-density area (HDA) of Zimbabwe.

## Methods

### Study design and setting

We conducted a cross-sectional study in Hatcliffe, a high density area (HDA), which has a population of close to 50,000 residents. Hatcliffe is the second fastest growing HDA in Harare. Most of the built up residential stands in Hatcliffe belong to retired persons that are predominantly poor. Many of the residents in Hatcliffe cannot afford their own homes hence they resort to renting. It is therefore common in Hatcliffe to find more than two families residing in one household. Most residents that reside in Hatcliffe and have jobs are engaged in informal economic activities in the central business district. There is no fast food outlets in the area. Poor households resort to urban agriculture for part of their food stuffs.

Hatcliffe is 25 km from Harare and separated by a road from the affluent low-density suburb of Borrowdale. Hatcliffe is surrounded by affluent low density suburbs. It has one clinic serviced by nurses and managed by the city council. ‘Old Hatcliffe’, a section of Hatcliffe, is tarred, has running water and is the oldest area. ‘Cooperative’ is a relatively new section of Hatcliffe which is not tarred and does not have running water.

### Study population and sample

In this study we intended to match the sexes in the households. We therefore selected two consenting adults; a male and female, aged 18 years and above, from every fourth household in the ‘Old Hatcliffe’ and ‘Cooperative’ area of Hatcliffe. The recruitment was done from the 21st of September 2018 to the 4th of October 2018′. The participants that we selected from these households had to meet the selection criteria of the study. We excluded all vulnerable people such as pregnant women, the mentally unstable and those that were bed-ridden at the time of the study. Those who had not lived in Hatcliffe for six months and above were also excluded. The prevalence of HTN and T2DM in Hatcliffe was not known. To achieve the minimum sample size of participants to be interviewed with an error margin of 5% and a 95% confidence interval, we used the following formula: N = Z^2^ P (1-P)/e^2.^ Where:

N is the population size.

Z is the level of confidence.

P is the population proportion.

e is the margin of error.

### Study instruments and data collection

The questionnaire administered for the face to face interviews was adapted from the WHO Stepwise Surveillance (STEPS) [17]. The STEPS questionnaire is a flexible and standardized tool which is used to monitor NCD risk-factor developments [17] . The questionnaire consisted of six sections. Section A of the questionnaire focused of biographical data, section B focused on behavioral data such as alcohol, salt and oil uptake and also questions on physical exercises were included. Section C focused on history of blood pressure, T2DM and cholesterol. Section D focused on physical measurements to determine the body mass index (BMI); section E focused on the biochemical measurements and section F, the health seeking behavior questions. The questionnaire was loaded into the KoBo Toolbox for Android (Kobo Toolbox, Harvard Humanitarian Initiative, and Cambridge, USA, available at: https://www.kobotoolbox.org/). We pretested the questionnaire using inpatients at the Hatcliffe Clinic for suitability. Data collection was conducted by five specifically trained community mobilizers who were familiar with the area.

### Data analysis

Data was exported from KoBo and checked for consistency and missing entries in an excel spreadsheet before being analyzed in STATA Version 15. Comparisons of knowledge/awareness of HTN and T2DM by sex where conducted reporting a *p* value using a student t-test for proportions. For significance differences between males and females *p* values of less than 0.05 were noted. A chi squared test for association between awareness of HTN status and behavioral practices was conducted reporting prevalence odds ratios and p values on the significance of the measure of association.

### Ethics statement

This study was granted permission by the Medical Research Council of Zimbabwe (A/2281) and the Biomedical Research Ethics Committee (BE628/17) of South Africa. Informed Consent Forms (ICF) were read out to the participant in Shona or English before the commencement of the interview. We also informed the participants that their names would not appear on the questionnaire. The participants consented by appending the signatures on the ICF. Participants who tested positive for either HTN or T2DM were referred to the clinic or the diabetologist who is part of the study team.

## Results

In this study we disaggregated the variables to see if there are any differences by sex as shown in Table 1.

**Table 1:** Comparison of knowledge/awareness of hypertension and type 2 diabetes by sex

Variable	Total Sample	Male (*n* = 245)	Female (*n* = 247)	*P*-value
Do you know if you have hypertension?				
Yes	56(11.4)	20(8.2)	36(14.6)	0.026
No	436(88.6)	225(91.8)	211(85.4)	
Do you voluntarily measure blood pressure regularly?				
Yes	41(8.3)	15(6.1)	26(10.5)	0.039
No	451(91.7)	230(93.9)	221(89.5)	
Do you know if you have T2DM?				
Yes	12(2.4)	5(2.0)	7(2.8)	0.562
No	480(97.6)	240(98.0)	240(97.2)	
Do you voluntarily measure your blood glucose?				
Yes	10(2.0)	3(1.2)	7(2.8)	0.205
No	482(98.0)	242(98.8)	240(97.2)	
How often do you eat take (carry) in a week?				
Frequent	247	119	128	0.506
Infrequent	245	126	119	

Eighty eight percent (88.6%) of the participants in the study did not know if they were hypertensive or not as shown in Table 1. Of the 245 men, 91.8% reported that were not aware if they had HTN or not. On the other hand 85.4 of women were also not aware of their HTN status. More men were therefore less aware of their status. Only 2% of men were aware of their T2DM status compared to 7% of women.

In addition, 91.7% of these participants had never voluntarily checked for hypertension. Furthermore, 98% of the participants had never voluntarily checked their blood glucose.

Ninety point 7 % (90.7%) of the participants took two or three meals a day. There was no significant differences between males and females who frequently ate take (carry) away (*p* = 0.506: student t-test for proportions).

We also found that 53.5% of the participants always used oil to cook their meals.

In this study 11.4% of the participants were aware of their hypertension status. Table 2 shows variables that are significantly associated with awareness of hypertension.

**Table 2:** Variables that are significantly associated with awareness of HTN

Variable	Awareness of Hypertension status		Odds Ratio	Chi- square *p*-value
	No(***n*** = 436)	Yes(***n*** = 56)		
Frequency of salt added to food				< 0.001
Never	37	14	1	
Sometimes	254	32	0.333	
Always	145	10	0.18	
Perception of salt amount taken per day				< 0.001
Minimum	91	27	1	
Adequate	277	24	0.25	
Excessive	68	5	0.29	
Addition of salt to already cooked meals				0.025
Never	112	6	1	
Sometimes	51	10	3.66	
Always	273	40	2.74	
Frequency of consumption of processed foods				
Never	37	9	1	0.015
Sometimes	247	37	0.62	
Always	152	10	0.27	

### Chi-square test for association between awareness of hypertension status and behavioral practices

This study shows that those who always add salt to their food where 82% less likely to be aware of their HTN status. Those who perceived to take excessive amounts of salt were 71% less likely to be aware of their HTN status. The study also reveals that those who always consume processed food were 73% less likely to be aware of knowing if they had HTN or not. Out of the 436 participants who were not aware of their HTN status, 62.6% of them reported that they had a habit of adding additional salt to food that had already been cooked.

There was also significant association shown between whether one knew their T2DM status and number of meals taken per day (*p* = 0.001).

Of importance to note is that those that indicated that they sometimes add salt to already cooked food were at least three times at risk of being hypertensive as compared to those who did not add.

## Discussion

The majority of the participants were not employed and were not aware of their HTN and T2DM statuses. Furthermore the participants relied on cheap high calorie, salty foods with very little nutritional value. Such faulty diets are risk factors for HTN and T2DM. These findings are consistent with Laraia assertion that the poor from developing countries have fewer nutritional food choices [18]. Murendo, reported that the Zimbabwean population has ‘poor dietary diversity’ [19].

The majority of the study participants did not aware if they had either HTN or T2DM. Sub-Saharan Africa has a high number of undiagnosed HTN and T2DM [20, 21]. The low figures of participants not testing for HTN or T2DM may imply that either the services are not accessible or they have no knowledge of them [22].

In this study females were more aware of testing for HTN and T2DM. This is consistent with PURE studies by Chow et al. that demonstrated that women were more aware of their hypertension status than their male counterparts [23]. Mufunda suggested that women tend to seek for information and self-care more than men [24] and further asserted that men have poor health-seeking behaviour and are risk-takers with regards their health compared to their female counterparts [24]. Studies by Bello –Oboist et al. have shown that there was a higher prevalence of HTN and T2DM females than males [25]. There is need for stakeholders to increase programs aimed at males to test for HTN and T2DM.

Our study found that many of the study participants add salt to meals already cooked with salt. In a study by Bhattacharya it was found that most of the participants were not aware of the daily recommended daily allowance of salt [26] .Bhattacharya further asserted that macro level factors (cultural practices, the level of education and measurements for salt used by cooks when preparing food) are important to consider in order for salt consumption to be reduced [26]. In a study in Malawi it was shown that salt consumption in the households in the urban areas was high [27]. Households in Zimbabwe prepare most meals using gravy thickeners and flavor cubes which already contain salt. Men especially in urban Zimbabwe have developed a leisure culture of barbequing where large amounts of salt are consumed. Most of the participants in this study gauged the quantity of salt in food by tasting.

There is limited scientific evidence suggesting side effects of excessive consumption of vegetable oil. However a study by Harris suggests that consumption of vegetable oils that are high in omega 6 fatty acid causes cardiovascular diseases [28]. In this study the majority of the participants always used vegetable oil for cooking. There is an increase in use of vegetable oil in SSA [29] .However, Forouhi suggested that some plant oils have beneficial properties in the context of T2DM although these are under-researched [30]. Vegetable oils have become more affordable, accessible and are perceived to improve the flavor of food [31]. Preparing meals with vegetable oil is becoming habitual; meals cooked without vegetable oil are perceived to be lacking in appearance. Olive oil is the healthier oil option but is costly and therefore not affordable [32]).In her study Nyeppi et al. reported an increase in vegetable oil use in southern African countries largely arguing that the people do not have other traditional options to use [33].

To the best of our knowledge our study is the first that has been carried out in a Zimbabwean HDA to ascertain awareness of salt and cooking oil usage with regards to HTN and T2DM. Therefore this study could aid in designing of national guidelines, programs and implementation of interventions.

## Conclusion

Our study established that many people in Hatcliffe have limited awareness of HTN and T2DM. Furthermore it was apparent that many study participants were not aware of the need and importance of regularly testing for HTN and T2DM so that they would know their status. The study shows that more women were aware of their HTN and T2DM status than men. The majority of the participants in the study perceived that the salt they consume was the right quantity. It was shown in this study that the majority of the participants always use vegetable oil when preparing meals.

### Recommendation

The study found that the participants had faulty dietary habits thus we recommend to government that there should be dietary education and behaviour change communication. The government is encouraged to review its policies with regards to HTN and T2DM in the context of dietary habits. Gender specific health promotion programs, such as mobile health and increase in media coverage, targeting males in HDA teaching them about healthy habits and NCDs and must be designed and implemented’.

We also recommend that the primary health system be equipped with resources and trained cadres so that there is scaling up of screening of HTN and T2DM in HDA.

### Limitation

This study could have looked at the sodium content in the urine of the participants but could not due to funding constraints. The study was conducted in a poor resource setting where inhabitants of Hatcliffe are predominantly are poor which might affect the generalizability. A wider national study with more coverage involving participants from medium to low density areas should be conducted. The questionnaire was administered face to face which might lead to bias by the interviewers.

## Data Availability

Data for this study is available upon request from the corresponding author on reasonable request.

## Abbreviations

HAD: High density area
HTN: Hypertension
LMIC: Low to medium income country
NCD: Non-communicable disease
SSA: Sub-Saharan Africa
T2DM: Type 2 Diabetes Mellitus
WHO: World Health Organisation

## References

[1] Naseem, S., et al., Prevalence of non-communicable diseases and their risk factors at a semi-urban community, Pakistan. Pan Afr Med J. 2016;23(1):151–151. 10.11604/pamj.2016.23.151.8974 PMC4894738 27303569

[2] Melaku YA, Temesgen AM, Deribew A, Tessema GA, Deribe K, Sahle BW (2016). The impact of dietary risk factors on the burden of non-communicable diseases in Ethiopia: findings from the global burden of disease study 2013. Int J Behav Nutr Phys Act.

[3] Sookram C, Munodawafa D, Phori PM, Varenne B, Alisalad A (2015). WHO’s supported interventions on salt intake reduction in the sub-Saharan Africa region. Cardiovascular diagnosis therapy.

[4] Lachat C, Otchere S, Roberfroid D, Abdulai A, Seret FMA, Milesevic J (2013). Diet and physical activity for the prevention of noncommunicable diseases in low-and middle-income countries: a systematic policy review. PLoS Med.

[5] Popkin BM (2006). Global nutrition dynamics: the world is shifting rapidly toward a diet linked with noncommunicable diseases. Am J Clin Nutr.

[6] Pastakia SD, Pekny CR, Manyara SM, Fischer L (2017). Diabetes in sub-Saharan Africa–from policy to practice to progress: targeting the existing gaps for future care for diabetes. Diabetes Metab Syndr Obes: targets, therapy.

[7] Ranasinghe P, Pigera A, Ishara M, Jayasekara L, Jayawardena R, Katulanda P (2015). Knowledge and perceptions about diet and physical activity among Sri Lankan adults with diabetes mellitus: a qualitative study. BMC Public Health.

[8] van de Vijver S, Akinyi H, Oti S, Olajide A, Agyemang C, Aboderin I, et al. Status report on hypertension in Africa-Consultative review for the 6th Session of the African Union Conference of Ministers of Health on NCD’s. Pan Afr Med J. 2014;16(1). 10.11604/pamj.2013.16.38.3100 PMC3932118 24570798

[9] Trieu K, McMahon E, Santos JA, Bauman A, Jolly K-A, Bolam B (2017). Review of behaviour change interventions to reduce population salt intake. Int J Behav Nutr Phys Act.

[10] Lassen A, Trolle E, Bysted A, Knuthsen P, Andersen E (2018). The salt content of lunch meals eaten at Danish worksites. Nutrients.

[11] Muthuri SK, Oti SO, Lilford RJ, Oyebode O (2016). Salt reduction interventions in sub-Saharan Africa: a systematic review. PLoS One.

[12] Seedat YK (2015). Why is control of hypertension in sub-Saharan Africa poor?. Cardiovascular journal of Africa.

[13] Marshall JA, Bessesen DH (2002). Dietary fat and the development of type 2 diabetes.

[14] Thornton J. Eliminate “toxic” trans fats from food by 2023, WHO urges. BMJ. 2018;361. 10.1136/bmj.k2154 29764815

[15] Mufunda E, Ernersson A, Hjelm K (2018). Limited knowledge of diabetes in patients attending an outpatient diabetes clinic at a referral hospital in Zimbabwe: a cross-sectional study. Pan African Medical J.

[16] Mutowo MP, Mangwiro JC, Lorgelly PK, Owen AJ, Renzaho A (2015). Hypertension in Zimbabwe: a meta-analysis to quantify its burden and policy implications. World Journal of Meta-Analysis.

[17] Riley L, Guthold R, Cowan M, Savin S, Bhatti L, Armstrong T (2016). The World Health Organization STEPwise approach to noncommunicable disease risk-factor surveillance: methods, challenges, and opportunities. Am J Public Health.

[18] Laraia Barbara A., Leak Tashara M., Tester June M., Leung Cindy W. (2017). Biobehavioral Factors That Shape Nutrition in Low-Income Populations. American Journal of Preventive Medicine.

[19] Murendo C, Nhau B, Mazvimavi K, Khanye T, Gwara S. Nutrition education, farm production diversity, and commercialization on household and individual dietary diversity in Zimbabwe. Food Nutrition Research. 2018(62). 10.29219/fnr.v62.1276. 10.29219/fnr.v62.1276 PMC5965157 29849533

[20] Mushoriwa F, Townsend N, Srinivas S (2017). Knowledge, attitudes and perception on dietary salt reduction of two communities in Grahamstown. South Africa Nutrition and health.

[21] Mbanya JCN, Motala AA, Sobngwi E, Assah FK, Enoru ST (2010). Diabetes in sub-saharan africa. Lancet.

[22] Ojo TT, Hawley NL, Desai MM, Akiteng AR, Guwatudde D, Schwartz JI (2017). Exploring knowledge and attitudes toward non-communicable diseases among village health teams in Eastern Uganda: a cross-sectional study. BMC Public Health.

[23] Chow CK, Teo KK, Rangarajan S, Islam S, Gupta R, Avezum A (2013). Prevalence, awareness, treatment, and control of hypertension in rural and urban communities in high-, middle-, and low-income countries. Jama.

[24] Mufunda E, Albin B, Hjelm K (2012). Differences in health and illness beliefs in zimbabwean men and women with diabetes. The open nursing journal.

[25] Bello-Ovosi BO, Asuke S, Abdulrahman SO, Ibrahim MS, Ovosi JO, Ogunsina MA (2018). Prevalence and correlates of hypertension and diabetes mellitus in an urban community in North-Western Nigeria. Pan African Medical Journal.

[26] Bhattacharya S, Thakur JS, Singh A (2018). Knowledge attitude, and practice regarding dietary salt intake among urban slum population of North India. Journal of family medicine and primary care.

[27] Prynn JE, Banda L, Amberbir A, Price AJ, Kayuni N, Jaffar S (2018). Dietary sodium intake in urban and rural Malawi, and directions for future interventions. Am J Clin Nutr.

[28] Harris WS, Shearer GC (2014). Omega-6 fatty acids and cardiovascular disease: friend, not foe?.

[29] Kearney J (2010). Food consumption trends and drivers. Philos Trans R Soc Lond Ser B Biol Sci.

[30] Forouhi NG, Misra A, Mohan V, Taylor R, Yancy W (2018). Dietary and nutritional approaches for prevention and management of type 2 diabetes. Bmj..

[31] Vorster HH, Bourne LT, Venter CS, Oosthuizen W (1999). Contribution of nutrition to the health transition in developing countries: a framework for research and intervention. Nutr Rev.

[32] Misra A, Singhal N, Khurana L (2010). Obesity, the metabolic syndrome, and type 2 diabetes in developing countries: role of dietary fats and oils. J Am Coll Nutr.

[33] Nnyepi MS, Gwisai N, Lekgoa M, Seru T (2015). Evidence of nutrition transition in southern Africa. Proc Nutr Soc.

